# Assessing quality of life-shortening *Wolbachia*-infected *Aedes aegypti* mosquitoes in the field based on capture rates and morphometric assessments

**DOI:** 10.1186/1756-3305-7-58

**Published:** 2014-02-03

**Authors:** Heng Lin Yeap, Jason K Axford, Jean Popovici, Nancy M Endersby, Iñaki Iturbe-Ormaetxe, Scott A Ritchie, Ary A Hoffmann

**Affiliations:** 1 Pest and Disease Vector Group, Bio21 Institute and the Department of Genetics, University of Melbourne, Parkville, Victoria, Melbourne 3010, Australia; 2 School of Biological Sciences, Monash University, Clayton, Victoria, Melbourne 3800, Australia; 3 School of Public Health, Tropical Medicine and Rehabilitation Sciences, James Cook University, Smithfield, Queensland, Cairns 4878, Australia

**Keywords:** *Wolbachia*, *Aedes aegypti*, Size, Shape, Quality

## Abstract

**Background:**

Recent releases have been carried out with *Aedes aegypti* mosquitoes infected with the *w*MelPop mosquito cell-line adapted (*w*MelPop-CLA) strain of *Wolbachia.* This infection introduced from *Drosophila* provides strong blockage of dengue and other arboviruses but also has large fitness costs in laboratory tests. The releases were used to evaluate the fitness of released infected mosquitoes, and (following termination of releases) to test for any effects of *w*MelPop-CLA on wing size and shape when mosquitoes were reared under field conditions.

**Methods:**

We monitored gravid females via double sticky traps to assess the reproductive success of *w*MelPop-CLA-infected females and also sampled the overall mosquito population post-release using Biogent Sentinel traps. Morphometric analyses were used to evaluate infection effects on wing shape as well as size.

**Results:**

Oviposition success as assessed through double sticky traps was unrelated to size of released mosquitoes. However, released mosquitoes with lower wing loading were more successful. Furthermore, *w*MelPop-CLA-infected mosquitoes had 38.3% of the oviposition success of uninfected mosquitoes based on the predicted infection frequency after release. Environmental conditions affected wing shape and particularly size across time in uninfected mosquitoes, but not in naturally-reared *w*MelPop-CLA-infected mosquitoes. Although the overall size and shape do not differ between naturally-reared *w*MelPop-CLA-infected and uninfected mosquitoes, the infected mosquitoes tended to have smaller wings than uninfected mosquitoes during the cooler November in comparison to December.

**Conclusion:**

These results confirm the lower fitness of *w*MelPop-CLA infection under field conditions, helping to explain challenges associated with a successful invasion by this strain. In the long run, invasion may depend on releasing strains carrying insecticide resistance or egg desiccation resistance, combined with an active pre-release population suppression program.

## Background

The virulent maternally-inherited endosymbiotic *Wolbachia* strain, *w*MelPop-CLA [1], reduces lifespan [2,3] and strongly blocks dengue virus proliferation in the primary dengue mosquito vector, *Aedes aegypti*[4-6]. Replacing field mosquitoes with *w*MelPop-CLA-infected individuals could therefore reduce dengue transmission in the field. However, *w*MelPop-CLA has deleterious effects on its host including reduced viability of mosquitoes in quiescent eggs [3,7], reduced fecundity [3,8], reduced ability to blood-feed [9,10], and altered development [3,11]. This poses significant challenges for the spread of the *w*MelPop-CLA infection in the field [3,12], unless such deleterious effects can be overcome through combined modalities [13]. However, these deleterious effects by themselves might provide an effective method for population suppression [3,7] particularly in genetically isolated sites [14].

Following the successful invasion of mosquitoes with the less virulent strain of *Wolbachia*, *w*Mel in two isolated suburbs of Cairns, Northern Queensland, Australia in 2011 [15], field trials releasing *w*MelPop-CLA in two other regions were undertaken in 2012 [16]. These releases provided an opportunity to assess the success of *w*MelPop-CLA-infected females in sourcing an oviposition site and to assess morphometric traits to evaluate fitness of the released mosquitoes. This follows earlier work [11] showing that larger females tend to be more successful at finding oviposition sites, and suggesting that infected mosquitoes emerging in the field have similar sizes to uninfected mosquitoes, at least for the non-virulent *w*Mel strain.

We monitored fitness based on successful oviposition and morphology of *w*MelPop-CLA-infected mosquitoes during the first three weeks of the release, and also geometric morphology post-release at the transition from the dry to wet season. The following questions were considered. (1) Are there differences in the measurements of morphometric traits between field-destined and recaptured released *w*MelPop-CLA-infected females? (2) Is the frequency of successful ovipositing females that are infected and released comparable to the estimated release frequency? (3) Will naturally-reared infected mosquitoes have differing body size and shape in contrast to uninfected mosquitoes? We then use these findings to help interpret the outcome of the *w*MelPop-CLA releases in terms of whether the released females are surviving, mating, blood feeding and ovipositing, as well as the fitness of subsequent generations in the field. For this purpose, we employed two mosquito trapping systems: double sticky traps (DST) [17] to monitor ovipositing female mosquitoes at the beginning of the release and BioGent Sentinel traps (BGS) to monitor the adult mosquito population several months after release [18-20]. We obtained body size and shape measurements from mosquitoes caught in both types of trap.

## Methods

### Released mosquitoes

The mosquitoes came from releases undertaken in 2012 at Machans Beach near Cairns in northern Queensland [16]. Releases were undertaken with offspring of *w*MelPop-CLA-infected mosquitoes established in two semi-field cages (8.0 m × 9.0 m × 4.1 m) [21] following a similar procedure described in Hoffmann *et al.*[15] where uninfected males derived from the field are periodically released into the cage to counter the effects of inbreeding. Offspring are established from eggs laid by these females and released soon after emergence. Releases took place over a 13-week period at Machans Beach and involved around 10 females being released weekly per house. Fitness assessments for released individuals were based on the first 10-day period of the release when individuals from multiple releases would not have been captured in traps (see below). Although these releases ultimately did not result in the *w*MelPop-CLA infection becoming established in Machans Beach [16], the infection persisted for several months after releases were terminated and this provided an opportunity to investigate fitness of the release material as well as morphometric effects of the infection in mosquitoes reared under field conditions.

### Morphometric traits and fitness of released mosquitoes

One hundred DSTs were employed in fifty residential locations (two traps each) in Machans Beach in the first week of the mosquito release. The DSTs [17] were made up of two black containers: the bottom container held 1L of water and a lucerne pellet infusion [22], while the top was clipped to the bottom and contained a sticky panel (UVR-32, Atlantic Paste and Glue) on the internal surface and a gap on top to allow mosquito entry. As a DST contains an infusion attractive to *Aedes*, up to 99% of the trapped *A. aegypti* consist of gravid/parous females [23]. Virgin gravid females are less receptive to oviposition site seeking [24,25], and females caught in DSTs were, therefore, assumed to be mostly mated. Samples were collected on 4th, 6th, 9th, 11th, 13th and 16th January 2012 and tested for *Wolbachia* infection.

Measurements of wing centroid size, thorax length and the wing size to thorax length (wing/thorax) ratio were compared among samples involving field-caught released mosquitoes from the DSTs, infected mosquitoes from the field cage and uninfected mosquitoes from the DSTs. Coefficients of variations (CoV) associated with the three morphometric measurements and wing shape (see below) were also compared.

### Comparing ovipositing infected females with estimates based on release frequencies

As releases of infected mosquitoes began on 4th January 2012 and these mosquitoes had not been blood-fed, *w*MelPop-CLA-infected females would not have been gravid on 4th January and 6th January. Females require approximately 2 to 3 days at 26–28°C to develop mature eggs post-blood feeding prior to oviposition [24,26,27]. Thus, only infected females caught after these dates were considered.

Assuming an equal and constant rate of trapping of non-gravid females for both uninfected and *w*MelPop-CLA-infected females, we estimated the infection frequency of ovipositing females directly attributed to the first release. We estimated the proportion of ovipositing infected females in the first release using data from collections on 9th, 11th and 13th January, prior to females from the second release contributing to traps.

We also estimated the expected number of mosquitoes in their first gonotrophic cycle from the first release to be caught in the traps after 13th January. This provided an estimate of the proportion of successfully ovipositing females within the first 10 days of release (4th-13th January). We did this by fitting two models. In the first, we fitted an exponential decay function on the daily trapping rate of *w*MelPop-CLA-infected females with respect to time. This was done via linear regression of the natural log of the daily trapping rate against the mid-point date between collection dates, excluding the first two, and the last collections. The second model was a logistic growth model on the cumulative number of mosquitoes trapped over time.

We assumed negligible contribution from the second gonotrophic cycle due to reduced survival of the *w*MelPop-CLA-infected mosquitoes [2]. Released *w*Mel-infected females are likely to have a field survival of 70-90% per day [28] and around 5-30% are expected to survive to the second gonotrophic cycle (10–15 days old). The survival rate is expected to be somewhat lower for *w*MelPop-CLA-infected females.

### Effects of infection on size and shape in generations subsequent to release

To examine the effect of the infection on traits in naturally-reared mosquitoes, samples from November-December 2012 were collected to study size and shape of mosquitoes at a time when the population remained polymorphic for the *w*MelPop-CLA infection, but well after releases had been terminated. BGS-traps were placed at around 100 residential properties with the consent of the owners. Traps were inspected once a week. BGS-traps are effective at capturing all adult stages of *A. aegypti* mosquitoes including young nullipars [18-20].

Wing size and shape were compared in mosquitoes from the BGS-traps; it was not possible to obtain thorax measurements because these were used immediately to screen for *Wolbachia* infection. Comparisons were made for wing size, CoV and shape between uninfected and infected groups, and also between collections. We used the total number of mosquitoes caught in the traps as an indicator of mosquito density and also noted the average temperature during the 20 days leading up to trap collection.

### *Wolbachia*-infection status

The DST samples were tested using the protocol detailed in Lee *et al.*[29] (see also Additional file 1: Table S1). PCR conditions proceeded with the following settings: 95°C for 10 minutes, 40 cycles of 95°C for 5 seconds, 58°C for 15 seconds and 72°C for 15 seconds, ending with a 95°C 1-minute heating followed by cool down to 40°C for 20 seconds before raising to 65°C. A melting curve analysis was performed immediately via a gradual increase of temperature from 65°C to 95°C. Using a Light Cycler 480 (Roche Applied Science), we determined the crossing point (Cp) values and melting temperatures (Tm) which allows us to determine presence or absence of *Wolbachia*. The *Wolbachia* specific primers in Lee *et al.* were replaced with *w*MelPop-CLA specific primers initially developed to screen the BGS-trap samples (see below).

The BGS-trap samples were screened using a different protocol. Mosquitoes collected in BGS traps in North Queensland were kept in 70% ethanol and shipped to Monash University, Melbourne for PCR. For DNA extractions, adult mosquitoes were washed in milli Q (Millipore) water and individually transferred to 96-well PCR plates containing a 2 mm glass bead and filled with 50 μL extraction buffer per well. The extraction buffer consisted of 4.8 mL squash buffer (10 mM Tris pH 8.2, 1 mM EDTA, 50 mM NaCl) and 60 μL proteinase K (15 mg/ml, QIAGEN). Each extraction plate included *w*Mel, *w*MelPop-CLA and uninfected mosquitoes from laboratory colonies as controls. After homogenizing the samples in a Mini-Beadbeater (Biospec Products) for 1.5 min, the plates were incubated in a 96-well thermocycler block for 5 min at 56°C then boiled at 95°C for 5 min (to inactivate proteinase K) and cooled down to 4°C. Extracted DNA was then stored at 4°C for up to 3 days prior to qPCR.

The primers and probes are described in Additional file 1: Table S1. The *A. aegypti* primers and probe sequences were designed for the *rps*17 gene [4]. The primers and probes specific for *w*Mel and *w*MelPop-CLA strains were designed using the PrimeTime qPCR tool (Integrated DNA Technologies). The *w*Mel specific primers and probe were designed to target the *WD0513* gene that is present in *w*Mel [30] but is absent in the *w*MelPop-CLA genome that was transinfected into the PGYP1 mosquitoes (Woolfit, unpublished results). The primers and probe specific for *w*MelPop-CLA were designed across the region spanning the *IS5* element inserted into *WD1310*[31] (and Riegler *et al.*, unpublished data), since this *IS5* insertion is absent in the *w*Mel *WD1310* gene [30]. Each of the three probes was labelled with a different fluorophore with non-overlapping emission wavelengths in order to be used in the same multiplex reaction: *rps*17 with 6-carboxyfluorescein (FAM), *IS5* with hexachlorofluorescein (HEX) and *WD0513* with cyanine5 (Cy5). The FAM and HEX labelled probes were quenched using the black hole quencher 1 (BHQ1) while the Cy5 probe was quenched with BHQ3.

Amplification and detection were completed on a LightCycler 480II system equipped with a 96- or 384-well block (Roche, Germany). The qPCR reaction was performed as follows: an initial denaturation step at 95°C for 5 min followed by 45 cycles at 95°C for 10 sec, 60°C for 15 sec and amplification at 72°C for 1 sec with single fluorescence acquisition. The reaction was prepared in 10 μL using 1 μL of DNA, 5 μL of LightCycler 480 Probe Master (Roche, Germany) and the volumes of primers and probes listed in (Additional file 1: Table S1). Fluorescence was acquired simultaneously for the 3 fluorophores used: FAM, HEX and Cy5. Data were analysed with the absolute quantification module using the second derivative maximum algorithm of the LightCycler480 II. Each PCR plate included known infected and uninfected control samples. The qPCR assay was able to distinguish between the *w*Mel and *w*MelPop-CLA strains with 100% accuracy on hundreds of analysed larvae and adults.

### Wing morphometrics

**Figure 1:**
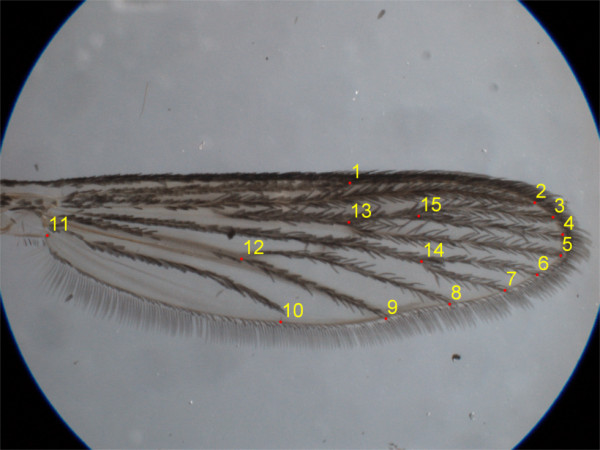
**Position and order of landmarks on *Aedes aegypti *** **wing.**

Left wings of mosquitoes (unless damaged) were mounted on a slide with Hoyer’s solution [32]. Each wing was photographed via 11.25× magnification with a Nikon SMZ1500 (Nikon Corporation, Shinagawa-ku, Tokyo, Japan) microscope and camera; photos were digitized with tPSUtil and tPSDig2 version 2.16 [33]. Fifteen landmarks were selected (Figure 1). Wing centroid size was used as a proxy for body size [34], computed as the square root of the sum of squares of the Euclidean distances between landmarks to the centroid. Landmarks were used for shape analyses (see below). Measurements of wing length were also taken between the alular notch (landmark 11) and the furthermost tip of the wing (approximately at landmark 4).

Repeatability of the landmarks was tested through repeat measurements on 200 wings. We ran a one-way analysis of variance (ANOVA) for each *x*-, *y*- coordinate and centroid size treating individuals as a fixed factor. The error then estimates within-individual variation (i.e. repeatability of measurements) [35]. Repeatability can then be computed as the ratio of among individual variance to the sum of variance of among and within individual variance. We obtained a repeatability of > 0.99 for all landmarks which is adequate for proceeding with an analysis of shape [35].

### Statistics

All size data were analysed in R3.01, while all shape analyses were performed in MorphoJ, but graphed in R. Wing centroid size and thorax length data were tested for normality using Shapiro-Wilks tests. Variances were compared to test for deviations from homoscedasticity. Pairwise-differences in means were tested via student *t*-tests, or unequal variance Welch *t*-test or non-parametric Mann–Whitney *U* tests, depending on whether there was unequal variance between groups and/or a breach in normality assumptions in one or both groups. ANOVA was only performed when data were normally distributed and groups had equal variances. CoVs were compared following Miller [36]. For analyses that required multiple comparisons, *p*-values were adjusted via the Dunn-Šidák procedure.

Procrustes superimposition was first performed on landmark coordinates to standardise wings to one unit centroid size, to remove orientation and location effects [37,38]. Covariance matrices were generated for each superimposed dataset to allow exploration of variation via Principal Component Analyses (PCA). To maximise separation between groups of interest, we used canonical variate analysis (CVA). If within group covariance matrices were very different between groups or only two groups are considered, pairwise group separation was performed via discriminant function analysis (DFA). Pairwise comparison tests were performed via permutation tests on Procrustes and Mahalanobis distances. Procrustes distance is defined as the square root sum of squares of the Euclidean distance between the individual Procrustes superimposed shape when compared to the superimposed average shape. Mahalanobis distances are the square root of the distances squared between the superimposed individual to the mean shape that are standardised by the covariance matrix of the distance variables. In situations where the *p*-value differed drastically due to anisotropy of the variation within groups and other factors, we took a conservative approach in evaluating the statistics.

Daily trapping rates across time of collection in DSTs were compared via Kruskal-Wallis tests (non-parametric ANOVA), with Mann–Whitney *U* tests used for all pairwise comparisons.

## Results

### Morphometric traits and fitness of released mosquitoes

Infected mosquitoes caught in the field within 10 days after the first release did not differ significantly from semi-field cage reared mosquitoes for wing centroid size (CS) or thorax length (TL) (see Table 1 for data, CS: *t* = 0.9838, df = 239, *p* > 0.32; TL: *t* = 1.4278, df = 263, *p* > 0.15). Wing length (WL) was also not significantly different between the two groups (field cage *vs* field caught: 2.95 *vs* 2.94 mm, Mann–Whitney *p* > 0.45). However, wing/thorax ratio was significantly different (see Table 1 for data, Mann–Whitney *U, p* = 0.043); field cage females had a lower wing/thorax ratio, i.e. higher wing load than those recollected from the field, though they are a subset of the same population. Coefficients of variation for all three morphometric traits were similar (*Z* < 1.9, *p* > 0.05).

**Table 1:** **Sample size, *n*, mean/median, standard deviation, SD, and coefficient of variation, CoV of morphometric traits by groups of *Aedes aegypti *** **from double sticky traps and field cage**

	**Wing centroid size (mm)**				**Thorax length (mm)**				**Wing size/thorax ratio**			
	***n***	**Mean**	**SD**	**CoV**	***n***	**Mean**	**SD**	**CoV**	***n***	**Median**	**SD**	**CoV**
**Field cage**	91	3.211	0.143	4.46%	100	1.468	0.070	4.74%	91	2.180	0.047	2.16%
**Cairns Jan 2012**												
**Infected (1)**	92	3.206	0.152	4.75%	97	1.465	0.080	5.43%	92	2.194	0.054	2.47%
**Infected (2)**	150	3.192	0.148	4.64%	165	1.454	0.078	5.35%	150	2.199	0.057	2.59%
**Negative**	245	2.827	0.294	10.39%	286	1.265	0.157	12.39%	244	2.242	0.089	3.99%

Infected (1) excludes *w*MelPop-CLA-infected females caught on 16 January because the excluded date would have included individuals from the second release.

Infected (2) includes all females trapped during the DST trapping period.

**Figure 2:**
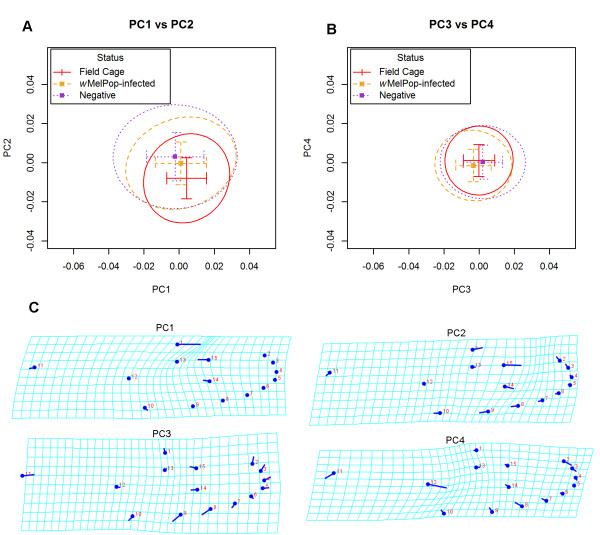
**Shape variation in field uninfected, infected field cage and infected field released *A. aegypti.*** Principal components showing the variation in shape for which PC1 explained the greatest variation followed by PC2, PC3, PC4 and so on. **(A)** PC1 *vs.* PC2 and **(B)** PC3 *vs.* PC4. Means and standard deviations of each group and ellipse outlining 90% of data, showing the high degree of overlap in variation for all groups within the double sticky trap (DST) and field cage data. Both *w*MelPop-CLA-infected and uninfected mosquitoes were caught in DSTs. **(C)** Shape variation in PC1, PC2, PC3 and PC4. The procedure over-exaggerates the variation, thus the shape variations were scaled down 10 times.

Since released infected mosquitoes were reared under high nutrition, we expected released *w*MelPop-CLA-infected female mosquitoes to be larger than uninfected female mosquitoes. This is reflected in wing size and thorax length (Table 1), including wing length, as comparisons were significant (CS: Welch-*t* = 16.36, df = 381.1, *p* < 0.001; TL: Welch-*t* = 17.10, df = 440.7, *p* < 0.001; WL: Mann–Whitney *U*, *p* < 0.001). Wing/thorax ratio (Table 1) of infected females also differed from uninfected females (Mann–Whitney *U*, *p* < 0.001), with infected females having a higher wing load than uninfected females. The coefficient of variation (CoV) was significantly lower (less than half in the case of the size traits) in infected females when compared with uninfected females (*Z* > 5, *p* < 0.001) as expected since field-emerging mosquitoes would have been reared under a range of conditions affecting size.PCA on shape landmarks for all DST and field cage data showed an even distribution across the first four principal components (PC) accounting for 62.4% of total shape variation (Figure 2). Around 25% (PC1) of variation can be explained by landmark 1 changing in the opposite direction to landmark 14 and 15; 16.5% (PC2) of variation is characterised by landmark 1, 14 and 15 tending towards the right as the outer landmarks tend towards the left; 12.6% (PC3) of variation describes an expansion of outer landmarks relative to inner landmark; 8.1% (PC4) of variation is explained by landmark 12 moving closer to the other inner landmarks while landmarks 7 to 10 move closer inwards.

**Figure 3:**
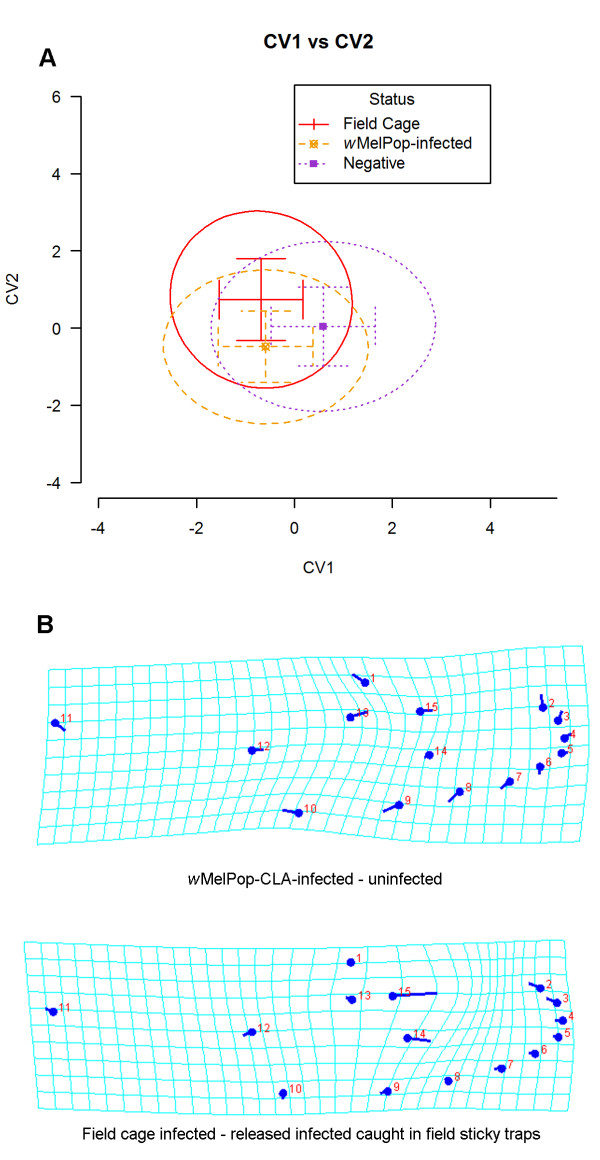
**Shape differentiation between field uninfected, infected field cage and infected field released *A. aegypti.*** Canonical variate analysis (CVA) treating infection status from DSTs and field cage as groups. **(A)** Canonical variate plot of mean and standard deviation, including 90% data ellipse for each group using first and second canonical variates (CV1 and CV2) that explains the greatest differentiation between groups. CV1 explains 61.9% of variation among groups while CV2 explains 29.8%. **(B)** Shape changes from *w*MelPop-CLA-infected females in DSTs to uninfected females in DSTs, and shape changes from field cage females to sticky trapped *w*MelPop-CLA-infected females. All shape changes were magnified 10 times for easier visualization.

A CVA (Figure 3) with DST infection status and field cage treated as separate groups indicated some difference between infected and uninfected caught in the field based on the first canonical variate (CV1). Along CV2, infected females caught in the field were differentiated from field cage females. These differences were statistically significant by permutation tests (10000 replicates) on Mahalanobis and Procrustes distances between groups (*p* < 0.0001).

Across time there were few changes in the measured morphometric traits. Data were separated by infection status because of significant heteroscedasticity among groups, involving a relatively higher variance in uninfected mosquitoes. Among the uninfected females, there was no evidence of differences for all morphometric measurements (CS: *F*_5, 239_ < 0.55, *p* > 0.74; WL: *F*_5, 239_ < 0.51, *p* > 0.77; thorax: *F*_5, 280_ < 0.32, *p* > 0.9; wing/thorax ratio: Mann–Whitney *U* statistics for pairwise comparisons, *p* > 0.05). Based on all pairwise comparisons using Mann–Whitney *U* statistics, infected females in DSTs also did not exhibit changes in any of the morphometric measures (*p* > 0.05). Pairwise comparison tests of Mahalanobis and Procrustes distances revealed no shape differences with time of collection, except in the case of infected females from 9 January and 16 January collections (*p* < 0.001).

### Assessing oviposition success of released, infected female

**Figure 4:**
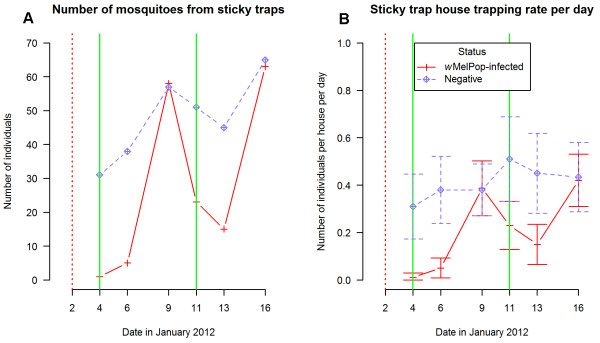
**Number of females trapped and associated trapping rate in DSTs. (A)** Total number of females caught in DSTs over six collections (4th, 6th, 9th, 11th, 13th and 16th January). **(B)** Female trapping rate for each house per day for all six collections with 95% confidence intervals (number of houses = 50). Both graphs were separated into uninfected and infected individuals. Red dotted vertical lines indicate day when DSTs were first deployed while green vertical lines are the first two releases of *w*MelPop-CLA-infected mosquitoes in Machans Beach.

A total of 165 *w*MelPop-CLA-infected females and 287 uninfected females were trapped from 4th January to 16th January and these mosquitoes provided an opportunity to evaluate the oviposition success of infected females before females from the ensuing release were captured. The rate of capture of uninfected females for each house on a per day basis was stable (Kruskal-Wallis χ^2^ = 4.08, df = 5, *p* > 0.54). On 9 January, there was a peak in infected females captured in the DSTs, with numbers comparable to those for uninfected females (Figure 4). The number and rate of trapped infected females dropped substantially before moving up again on 16th January as a result of the second release on 11 January. After excluding data for the 4th, 6th and 16th January (infected mosquitoes that were not ovipositing or not from the first release), traps caught 96 infected females *vs.* 222 uninfected females, or around 30% of the ovipositing females were infected.

We estimated possible contributions of the first release to the DST collections beyond the 16th January by extrapolating from data collected 9th to 13th January. Based on a logistic growth model or exponential decay function, we estimated that on the 16th January there was a contribution of 1 (1%) or 9 (8.3%) *w*MelPop-CLA-infected mosquitoes from the first release, respectively (Additional file 2: Figure S1 and Additional file 3: Figure S2, Additional file 4: Supporting Text). Assuming indefinite mosquito survival, the two models predicted that 1 (1%) or 15 (13.8%) mosquitoes would have been caught in the first gonotrophic cycle after the 13th January respectively (Additional file 2: Figure S1 and Additional file 3: Figure S2, Additional file 4: Supporting Text). This means that 86-99% of gravid females in the first gonotrophic cycle will seek an oviposition site within the first 10 days of release (or before adult females reach an age of 13 days), or 52-60% within five days after release (before an age of 8 days).

### Effects of infection on size and shape in generations subsequent to release

BGS-trap data from November-December 2012 were separated by sex because males and females were significantly differentiated for wing size and wing length (males smaller than females: median CS 2.19 *vs.* 2.74 mm; Mann–Whitney *U*, *p* < 0.001) and shape (pairwise comparison of Mahalanobis and Procrustes distance, *p* < 0.0001). PCA yielded a first principal component, PC1, which showed a heterogeneous distribution of variation among sexes and which can be further differentiated via DFA (Additional file 5: Figure S3). The separation in shape is distinct enough (more than size) to show that there was an individual wrongly sexed as a female. PCA on female and male data indicated a homogeneous distribution of variance (not shown), with noticeable variation attributed to the separation of landmark 1 from landmark 14 and 15 in both PC1 and PC2.

The overall mean wing size of uninfected and naturally-reared *w*MelPop-CLA-infected mosquitoes was not significantly different for both males (CS: Means 2.20 *vs.* 2.16 mm, *t* = 1.22, df = 193, *p* > 0.22) and females (CS: Means 2.76 *vs.* 2.73 mm, *t* = 0.73, df = 179, *p* > 0.47). Coefficients of variation for wing size also did not differ between uninfected and infected mosquitoes for both males (CoV: 9.52% *vs.* 8.31%, *Z* < 1.14, *p* > 0.25) and females (CoV: 10.32% *vs.* 11.93%, *Z* < 1.27, *p* > 0.21). The same pattern was evident if wing length was considered. There were no differences in overall shape for both males and females between uninfected and *w*MelPop-CLA-infected mosquitoes when running permutation tests comparing Mahalanobis distance (Male: *p* > 0.38; Female: *p* > 0.29).

The amount of size variation measured by the CoV appears to fluctuate (See Table 2), but CoV was not significantly different within and among both infected and uninfected mosquitoes (separated by sex) (*Z* < 1.96, *p* > 0.05). Only the CoV of uninfected and infected females from the second collection was significantly different (*Z* > 1.96, *p* < 0.05) (see CoVs in Table 2).

**Table 2:** **Size measures and statistics of BGS-trap samples of *Aedes aegypti *** **separated by collection, sex and infection status**

		**Wing centroid size (mm)**								
		**Uninfected**				***w*** **MelPop-CLA-infected**				
**Sex**	**Collection**	***n***	**Mean**	**SD**	**CoV**	***n***	**Mean**	**SD**	**CoV**	**Comparison**
**Females**	**1**	16	2.96	0.27	9.16%	12	2.63	0.18	6.91%	*p* < 0.01
	**2**	31	2.70	0.24	9.06%	17	2.72	0.43	15.96%	NS
	**3**	23	2.88	0.27	9.40%	8	2.79	0.30	10.66%	NS
	**4**	25	2.74	0.28	10.07%	10	2.70	0.35	12.95%	NS
	**5**	32	2.65	0.27	10.39%	7	2.89	0.17	5.86%	0.05 > *p* > 0.01
**Males**	**1**	34	2.29	0.18	7.82%	18	2.19	0.16	7.46%	0.10 > *p* > 0.05
	**2**	35	2.21	0.21	9.42%	16	2.06	0.17	8.07%	0.05 > *p* > 0.01
	**3**	13	2.33	0.09	3.77%	9	2.19	0.16	7.46%	*p* < 0.01
	**4**	30	2.13	0.21	9.67%	5	2.29	0.26	11.20%	NS
	**5**	29	2.07	0.20	9.82%	6	2.15	0.13	5.88%	NS

Collections 1 to 3 are in November 2012, 4 and 5 are in December 2012.

*n*: sample size, SD: standard deviation, CoV: Coefficient of variation.

Wing size pairwise comparisons were made between uninfected and *w*MelPop-CLA-infected from the same collection. NS = Non significant, we also reported the *p*-value if less than 0.1.

We note that the average temperature in the 20 days before each trapping period was approximately 1.5-2.0°C higher in the two December sessions (27.5-29.5°C) than the three November sessions (25.8-27.9°C), so we decided to compare individuals grouped by month of trapping. In pairwise comparisons, uninfected mosquitoes trapped in November tended to be larger than those collected in December. Males from the first and third trapping session (7th and 21st November) were both significantly larger than males from 12th December and 19th December (*p* < 0.05 after correcting for multiple comparisons). Similarly, females from the first and third session (7 and 21 November) were both significantly larger than females from 19th December (*p* < 0.05 after correcting for multiple comparisons), although this was not the case for females from 14th November. However, on the 14th November the density was much higher; 130 mosquitoes were trapped *vs* 78–97 during the other trapping sessions. Conversely to uninfected mosquitoes, infected mosquitoes did not show any clear distinction in wing size and length between collections after accounting for multiple comparisons (all *p* > 0.05).

When comparing infected and uninfected mosquitoes from each collection, uninfected males were larger than infected males in the first three collections (in November, especially 14th and 21st) but there were no differences in the December collections (Table 2). Uninfected females were larger than infected females only in the first collection, and smaller than infected females in the last collection (Table 2). Here, we emphasise that there appears to be a trend for decreasing size between November and December in uninfected mosquitoes but no significant trend in infected mosquitoes.

**Figure 5:**
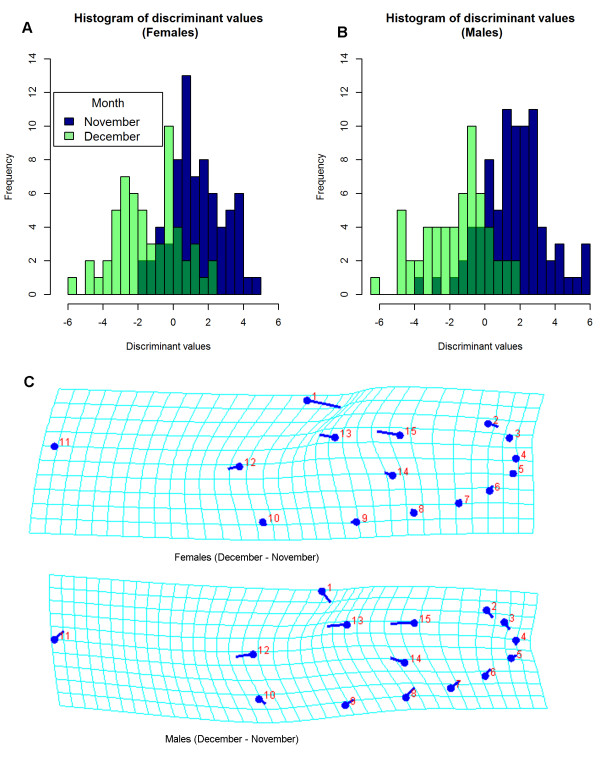
**Shape differentiation in uninfected mosquito caught in November and December. (A)** Discriminant value histogram for uninfected females comparing between months of BGS-trapping. **(B)** Discriminant value histogram for uninfected males. **(C)** Shape change for uninfected females from December (points) to November, and shape change for uninfected males from December to November. All shape changes were magnified 10 times.

Permutation tests on pairwise comparison of Mahalanobis and Procrustes distance for shape suggested differences between the three November collections and the two December collections for both uninfected males and females (0.05 > *p* > 0.0001). Infected males and females did not differ between collections for the same test. When collections were grouped into month of collection, both uninfected males and females showed significant difference in distances between November and December collections (*p* < 0.001) but not among infected individuals (*p* > 0.05). Landmark 1 was closer to landmark 13 and 15 in November uninfected mosquito samples (Figure 5). There was no clear evidence of differences in distances between uninfected and infected mosquitoes at any time point.

## Discussion

Released mosquitoes containing the *w*MelPop-CLA infection were found to be large with a low variance compared with uninfected mosquitoes from the field. A similar result was obtained with released *w*Mel-infected mosquitoes [11]. Within the released mosquitoes, there was no evidence for direct selection on size because size of field cage reared and captured mosquitoes were similar. Again, this is largely consistent with the results for *w*Mel [11] and may simply reflect the narrow variance in size of the released mosquitoes. Size also did not differ among collection time points, suggesting no bias for time to successful blood feeding.

In contrast, there was evidence that the wing/thorax ratio differed between the cage and field samples, suggesting that direct selection had occurred on this trait and/or that wing/thorax ratio had influenced ability to locate the DSTs. In insects, wing/thorax ratio can contribute to flight ability and dispersal. For instance, field releases of *Drosophila* showed that under field conditions, flies that have dispersed relatively further have a higher wing/thorax ratio [39]. In the case of released mosquitoes, individuals with a higher ratio (lower wing load) may achieve relatively greater success at locating breeding sites or exhibit a relatively higher survival. Despite average dispersal of *A. aegypti* being low and < 200 m [40,41], the ability to disperse is likely to be an important fitness determinant in behaviours such as "skip oviposition", host-seeking and oviposition site seeking [42-46]. The significant difference in shape may also be related to flight ability, but it is unclear if there are physical effects of subtle wing shape differences on flight ability.

The DST results suggest that about 30% of females caught were *w*MelPop-CLA-infected. This is substantially lower than the proportion of the population consisting of infected mosquitoes, which was estimated to be 53.0% (SA Ritchie *et al.*, unpublished) based on the approach outlined in [28]. These results suggest 38.3% successful oviposition in *w*MelPop-CLA-infected relative to uninfected females (Supporting Text). It thus appears that released *w*MelPop-CLA-infected females perform poorly on blood-feeding, female mating success, and/or oviposition site seeking in comparison to uninfected mosquitoes. Although this *Wolbachia* infection is known to cause severe age related fitness reduction [2,9,10], the cohort used in this estimation is relatively young (five to 13 days old), thus we cannot suggest that this is also age-related.

We estimated that most of the *w*MelPop-CLA-infected mosquitoes (86-99%) would have oviposited before 10 days after the release. This meant that most mosquitoes took a blood meal within 7 days after the release, suggesting a daily blood feeding success of 46-50%. Daily biting rate per mosquito in one study was approximated to 0.63-0.76 [47] but this accounted for multiple blood-feeds via histologic methods. Our estimates are similar to those obtained in previous DST studies on *Aedes*[45,48]. For instance, Marini *et al.*[48] suggested that 50-75% of marked-released, unfed *Aedes albopictus* females became gravid in the first five days after release.

The *w*MelPop-CLA-infected mosquitoes collected well after releases were terminated (i.e. subsequent generations breeding in the field) had a similar size, wing/thorax ratio and shape to uninfected mosquitoes. This is consistent with the absence of any substantial differences in naturally reared *w*Mel-infected mosquitoes when compared with uninfected field mosquitoes [11]. However, wing size in *w*MelPop-CLA-infected mosquitoes was relatively constant, whereas the wing size of uninfected mosquitoes generally decreased in response to an increase in density and/or especially temperature, as expected [49-52]. Laboratory experiments suggest that wing size of *w*MelPop-CLA-infected mosquitoes may not change much even when mosquitoes develop for longer and emerge late under high density conditions (PA Ross *et al.*, unpublished). As large size tends to be associated with higher fitness [11,53,54], *w*MelPop-CLA-infected mosquitoes may be at a particular disadvantage during periods where environmental conditions result in a longer larval development time (i.e. lower temperatures), because the costs of slow development are then not countered by a relatively larger size.

## Conclusions

The findings of this study suggest that *w*MelPop-CLA-infected mosquitoes have low fitness in the field. The lower oviposition success rate and smaller size of infected mosquitoes under some environmental conditions mean that the unstable equilibrium that has to be exceeded for successful invasion may be high for this infection even in the wet season [55]. In the dry season, additional fitness costs associated with egg quiescence are likely to increase the unstable point even further, and even if invasion is successful during the release, the infection may not persist [3,7] as was observed [16]. The lower wing/thorax ratio of released mosquitoes also suggests that dispersal ability may be limited in released mosquitoes. These effects on mosquito fitness and morphology were successfully detected using double sticky traps (DSTs) when coupled with mosquito monitoring based on BGS-traps. We recommend that future biocontrol releases using *Aedes aegypti* or related species should include a deployment of DSTs or the new gravid *Aedes* trap [56] for evaluating mosquito quality. This approach provides a rapid assessment of fitness under field conditions, which is likely to be more informative in predicting field performance than laboratory-based measures of fitness.

We also make a number of recommendations for future releases with this strain. (1) Release programs should aim to release a high frequency of *w*MelPop-CLA-infected mosquitoes (comprising >50% of the existing population). If high release numbers are not possible, successful invasion may depend on suppression of all life stages of the existing population before releases are initiated [57]. This will be particularly important when releases are undertaken in areas where estimated population sizes are large [58]. (2) Releases should take place over a long period of time. An extended release period helps to ensure that *Wolbachia* frequencies will exist over an unstable point for some time. (3) Nutritional regimes that lead to optimal wing/thorax ratios but maintain large adult size need to be explored. This will require a detailed understanding of the relationship between nutritional components and the asymmetric change in thorax length and wing size. By increasing the overall amount of food available, wing/thorax ratio will probably always decrease since thorax length appears to increase at a relatively faster rate than wing length with increased food [11]. Because of these factors, invasion of *w*MelPop-CLA is therefore only likely to occur in relatively isolated populations and/or when releases are tied to another modality, such as vector control to reduce the uninfected population, to assist in the spread of the infection [57]. The *w*MelPop-CLA strain may need to be modified through multi-generational artificial selection to alter traits such as egg desiccation resistance or insecticide resistance prior to release [13].

## Abbreviations

CLA: Mosquito cell-line adapted; DST: Double sticky traps; BGS: BioGents sentinel; FAM: 6-Carboxyfluorescein; HEX: Hexachlorofluorescein; Cy5: Cyanine5; BHQ: Black hole quencher; CoV: Coefficient of variation; PCA: Principal component analysis; CVA: Canonical variate analysis; DFA: Discriminant function analysis; CS: Centroid size; TL: Thorax length; WL: Wing length.

## Competing interests

The authors declare that they have no competing interests.

## Authors’ contributions

Designed the experiment: HLY, JKA, SAR, NME, AAH; Development of the *Wolbachia* screening protocol: JP, IIO; Performed the experiments: HLY, JKA; Performed the analysis: HLY, AAH; Contributed to screening, materials and equipment: IIO, JP, SAR, AAH; Wrote the paper: HLY, AAH. All authors read and approved the final version of the manuscript.

## Supplementary Material

Additional file 1: Table S1 Primers/probes and optimal concentrations. Click here for file

Additional file 2: Figure S1 Logistic growth model on cumulative number of *w*MelPop-CLA-infected females trapped. Cumulative number of infected female mosquitoes trapped in double sticky traps (DST) *versus* number of days after the first release. The estimate is based on numbers of infected females caught on 9th, 11th and 13th January 2012, with the first two time points (4th and 6th January) assumed to be close to zero. The estimated curve is most likely an underestimate as we lack data for any potential lag phase. Click here for file

Additional file 3: Figure S2 Exponential decay fitted on *w*MelPop-CLA-infected female mosquito trapping rate. Observed and estimated infected female daily trapping rate per house over time, based on rates for 9th, 11th and 13th January 2012. 50 houses were involved in this study. Click here for file

Additional file 4: Supporting Text Information on the calculation of number of expected females that will be caught in the first gonotrophic cycle beyond 13th January 2012 (9 days after the first release) based on the two models described in Figures S1 and S2. This is followed by the derivation of the relative oviposition success rate of released *w*MelPop-CLA-infected females to field uninfected females. Click here for file

Additional file 5: Figure S3 Shape differentiation between males and females. Discriminant values from discriminant function analysis of wing shape of BGS-trap mosquito samples based on sex. One 'female’ recorded a discriminant value of 18.48, which is an outlier compared to the other females and likely to be a misidentified individual. Click here for file
