# Supplementary material for: Assessing quality of life-shortening Wolbachia-infected Aedes aegypti mosquitoes in the field based on capture rates and morphometric assessments
Source: Parasit Vectors. 2014 Feb 3;7:58. doi: 10.1186/1756-3305-7-58 (PMC4015819; doi:10.1186/1756-3305-7-58)
Supplement: Additional file 1: Table S1 — Primers/probes and optimal concentrations. [file 1756-3305-7-58-S1.doc]

**Table S**1. Primers/probes and optimal concentrations

|  | Primer/probe | Sequences (5’-3’) | Trap | Volume (μL) | |
| --- | --- | --- | --- | --- | --- |
|  |  |  |  | DSO | BGS |
|  |  |  |  |  |  |
| Mosquito | mRpS6_F | AGTTGAACGTATCGTTTCCCGCTAC | DSO | 0.4 |  |
|  | mRpS6_R | GAAGTGACGCAGCTTGTGGTCGTCC | DSO | 0.4 |  |
| *A. aegypti* | aRpS6_F | ATCAAGAAGCGCCGTGTCG | DSO | 0.4 |  |
|  | aRpS6_R | CAGGTGCAGGATCTTCATGTATTCG | DSO | 0.4 |  |
|  | Rps17_F | TCCGTGGTATCTCCATCAAGCT | BGS |  | 0.25 |
|  | Rps17_R | CACTTCCGGCACGTAGTTGTC | BGS |  | 0.25 |
|  | Rps17_Probe | FAM-CAGGAGGAGGAACGTGAGCGCAG-BHQ1 | BGS |  | 0.1 |
| *w*Mel | WD0513_F | CAAATTGCTCTTGTCCTGTGG | BGS |  | 0.3 |
|  | WD0513_R | GGGTGTTAAGCAGAGTTACGG | BGS |  | 0.3 |
|  | WD0513_Probe | Cy5- TGAAATGGAAAAATTGGCGAGGTGTAGG-BHQ3 | BGS |  | 0.3 |
| *w*MelPop-CLA | IS5_F | CTCATCTTTACCCCGTACTAAAATTTC | DSO/BGS | 0.4 | 0.25 |
|  | WD1310_R | TCTTCCTCATTAAGAACCTCTATCTTG | DSO/BGS | 0.4 | 0.25 |
|  | IS5_Probe | HEX-TAGCCTTTTACTTGTTTCCGGACAACCT-BHQ1 | BGS |  | 0.25 |
|  |  |  |  |  |  |

Primer sets were used to screen either: DSO, double sticky trap and/or BGS, BioGent Sentinel Trap

Volume is 10 μM primer solution per 10 μL reaction (μL)

**References**

1. Lee SF, White VL, Weeks AR, Hoffmann AA, Endersby NM (2012) High-throughput PCR assays to monito*r Wolbach*ia infection in the dengue mosquito *(Aedes aegyp*ti) an*d Drosophila simula*ns. Appl Environ Microbiol 78: 4740-4743.
