# Supplementary figures and images for: Assessing quality of life-shortening Wolbachia-infected Aedes aegypti mosquitoes in the field based on capture rates and morphometric assessments
Source: Parasit Vectors. 2014 Feb 3;7:58. doi: 10.1186/1756-3305-7-58 (PMC4015819; doi:10.1186/1756-3305-7-58)

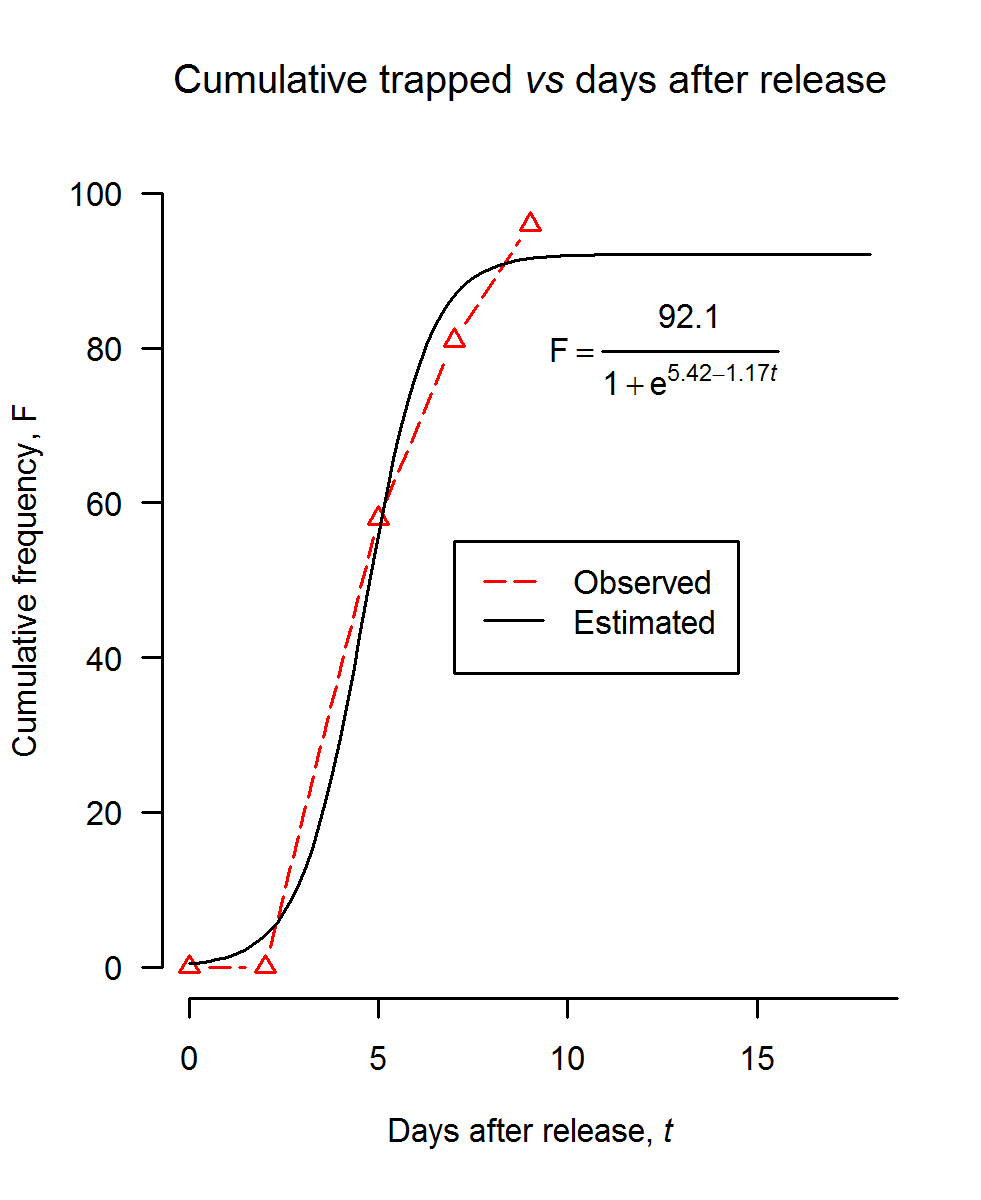

Supplement: Additional file 2: Figure S1 — Logistic growth model on cumulative number of wMelPop-CLA-infected females trapped. Cumulative number of infected female mosquitoes trapped in double sticky traps (DST) versus number of days after the first release. The estimate is based on numbers of infected females caught on 9th, 11th and 13th January 2012, with the first two time points (4th and 6th January) assumed to be close to zero. The estimated curve is most likely an underestimate as we lack data for any potential lag phase. [file 1756-3305-7-58-S2.tiff]

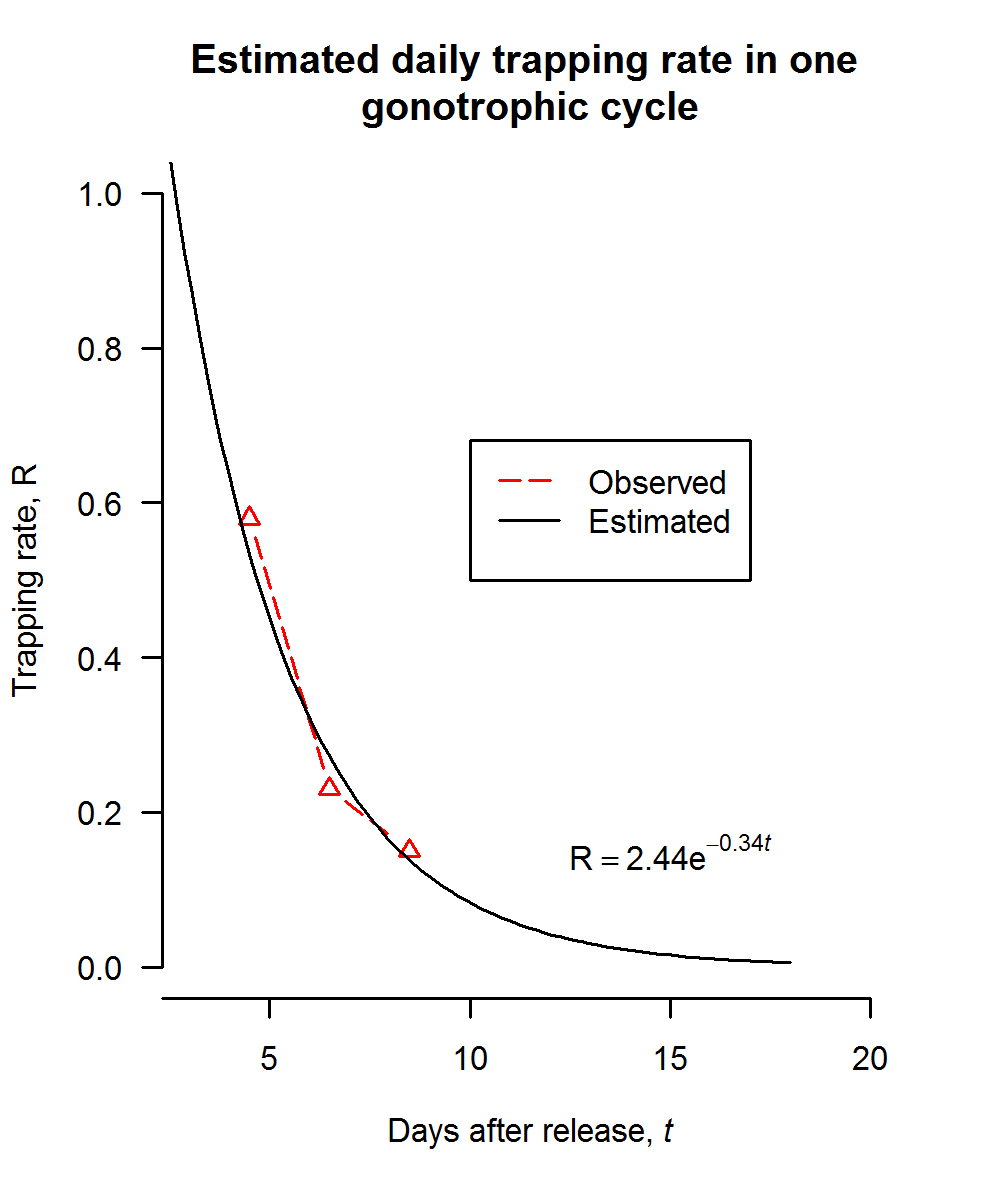

Supplement: Additional file 3: Figure S2 — Exponential decay fitted on wMelPop-CLA-infected female mosquito trapping rate. Observed and estimated infected female daily trapping rate per house over time, based on rates for 9th, 11th and 13th January 2012. 50 houses were involved in this study. [file 1756-3305-7-58-S3.tiff]

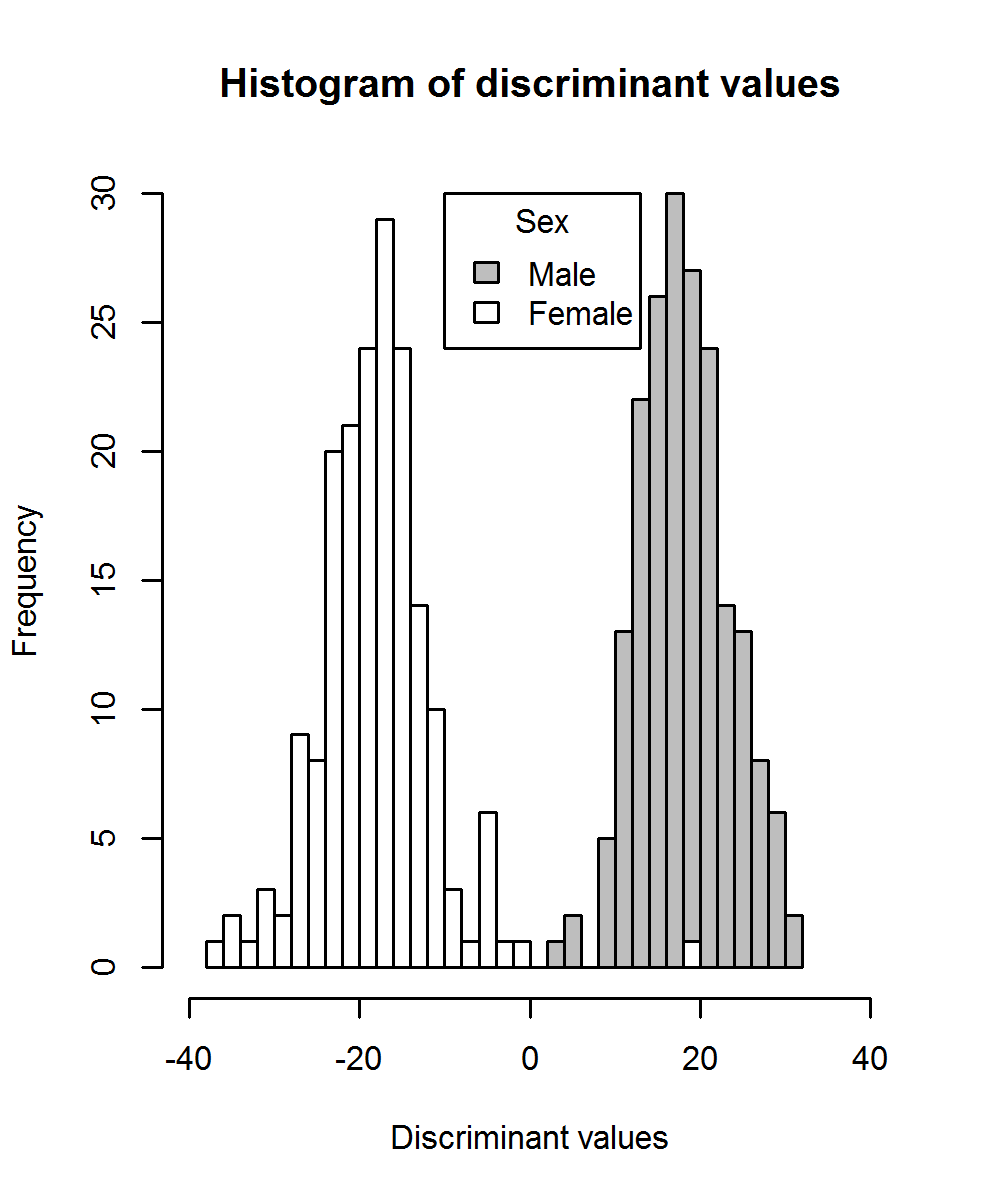

Supplement: Additional file 5: Figure S3 — Shape differentiation between males and females. Discriminant values from discriminant function analysis of wing shape of BGS-trap mosquito samples based on sex. One 'female’ recorded a discriminant value of 18.48, which is an outlier compared to the other females and likely to be a misidentified individual. [file 1756-3305-7-58-S5.tiff]
