# Supplementary material for: Assessing quality of life-shortening Wolbachia-infected Aedes aegypti mosquitoes in the field based on capture rates and morphometric assessments
Source: Parasit Vectors. 2014 Feb 3;7:58. doi: 10.1186/1756-3305-7-58 (PMC4015819; doi:10.1186/1756-3305-7-58)
Supplement: Additional file 4: Supporting Text — Information on the calculation of number of expected females that will be caught in the first gonotrophic cycle beyond 13th January 2012 (9 days after the first release) based on the two models described in Figures S1 and S2. This is followed by the derivation of the relative oviposition success rate of released wMelPop-CLA-infected females to field uninfected females. [file 1756-3305-7-58-S4.docx]

**Supporting Text** Information on the calculation of number of expected females that will be caught in the first gonotrophic cycle beyond 13 January 2012 (9 days after the first release) based on the two models described in Figures S1 and S2. This is followed by the derivation of the relative oviposition success rate of released *w*MelPop-CLA-infected females to field uninfected females.

**Logistic growth model on cumulative number of mosquitoes trapped, (see Figure S1):**

Estimated number of mosquitoes trapped on 16 January which is 12 days after the release

$$\left( \frac{92.1}{1+ e^{5.42-1.17t}} \right)_{t=9}^{t=12}\approx1$$

Estimated number of mosquitoes trapped beyond 13 January (beyond 9 days after the release)

$$\left( \frac{92.1}{1+ e^{5.42-1.17t}} \right)_{t=9}^{t\to\infty}\approx1$$

92.1 is the estimated total number of mosquitoes in their first gonotrophic cycle that will be caught in the trap based on this model over an indefinite amount of time.

**Exponential decay function on daily trapping rate per house (See also Figure S2):**

Estimated number of mosquitoes caught between 10 to 12 days after release (16 January)

$$=50*\sum_{t=10}^{12} 2.44e^{-0.34t}\approx9$$

Estimated number of mosquitoes caught after 13 January (beyond 9 days of release)

$$=50*\lim_{n\to\infty} \sum_{t=10}^{n} 2.44e^{-0.34t}=50*\frac{2.44e^{-0.34\left( 10 \right)}}{1-e^{-0.34}}\approx15$$

$$Given that \lim_{n\to\infty} \sum_{i=k}^{n} {ar}^{i}=\frac{{ar}^{k}}{1-r} for \left| r \right|<1, i\in Z$$

Total number of mosquitoes in their first gonotrophic cycle that will be caught in the traps based on this model is:

$$=50*\lim_{n\to\infty} \sum_{t=4}^{n} 2.44e^{-0.34t}=50*\frac{2.44e^{-0.34\left( 4 \right)}}{1-e^{-0.34}}\approx109$$

Mosquitoes caught before 4 days were unlikely to oviposit

**Determining the reduction in successful oviposition in infected mosquitoes relative to uninfected mosquitoes**

Let

*a* = Expected starting frequency of infection following the first release

*s_i_* = Proportion of successfully ovipositing *Wolbachia*-infected females

*s_u_* = Proportion of successfully ovipositing uninfected females

If *p*_i_ = Frequency of *Wolbachia*-infected ovipositing females

Then,

$$p_{i}= \frac{s_{i}a}{s_{u}\left( 1-a \right)+ s_{i}a}$$

$$\therefore s_{i}= \frac{s_{u}p_{i}(1-a)}{a(1-p_{i})}$$

Let *r_i_* = Relative successful oviposition in *Wolbachia*-infected relative to uninfected

$$r_{i}= \frac{s_{i}}{s_{u}}$$

$${\therefore r}_{i}=\frac{p_{i}(1-a)}{a(1-p_{i})}$$
